# Correlations of Biomarkers and Self-Reported Seafood Consumption among Pregnant and Non-Pregnant Women in Southeastern Louisiana after the Gulf Oil Spill: The GROWH Study

**DOI:** 10.3390/ijerph14070784

**Published:** 2017-07-14

**Authors:** Leah Zilversmit, Jeffrey Wickliffe, Arti Shankar, Robert J. Taylor, Emily W. Harville

**Affiliations:** 1 Department of Epidemiology, Tulane School of Public Health and Tropical Medicine, New Orleans, LA 70112, USA; eharvill@tulane.edu; 2 Department of Global Environmental Health Sciences, Tulane School of Public Health and Tropical Medicine, New Orleans, LA 70112, USA; jwicklif@tulane.edu; 3 Department of Global Biostatistics and Data Science, Tulane School of Public Health and Tropical Medicine, New Orleans, LA 70112, USA, sarti@tulane.edu; 4 Department of Veterinary Integrative Biosciences, Texas A&M University, College Station, TX 77843, USA; rtaylor@cvm.tamu.edu

**Keywords:** seafood consumption, mercury exposure, food frequency questionnaires, pregnancy, polyunsaturated fatty acids

## Abstract

Seafood contains health-promoting fatty acids, but is often contaminated with mercury (Hg), complicating recommendations and choices around fish consumption during pregnancy. Self-reported diet may be subject to inaccuracy and this inaccuracy could differ according to pregnancy status. We investigated correlations between self-reported seafood consumption and blood levels of Hg and n-3 polyunsaturated fatty acids (PUFAs) in women affected by the Deepwater Horizon oil spill. Spearman correlation coefficients were calculated comparing log blood Hg and n-3 PUFAs to seafood consumption, then stratified by pregnancy status. Crude and adjusted linear regression models were constructed using biomarkers of Hg and n-3 PUFA and seafood consumption, adjusting for age and pregnancy status. Weak but significant correlations were found between log Hg levels and intake of Hg-containing seafood (r = 0.15) and were slightly stronger among pregnant women (r = 0.22, vs. r = 0.10). Biomarkers for n-3 PUFAs were significantly correlated with seafood consumption (r = 0.12). Hg-containing seafood consumption was associated with increased blood level Hg in the highest quartile in both unadjusted (β = 0.34, 95% CI: 0.15–0.53) and adjusted models (β = 0.28, 95% CI: 0.08–0.48). Self-reported seafood consumption was correlated with biomarkers of both n-3 PUFA and Hg, but this association was different when stratified by pregnancy status. Pregnant women may have better recall of Hg-containing seafood compared to nonpregnant women.

## 1. Introduction

Seafood, including finfish (fish) and shellfish, may have healthy and unhealthy properties, especially during pregnancy. Polyunsaturated fatty acids (PUFA) have been associated with beneficial health outcomes such as a reduction in risk of cardiovascular disease, hypertension, atherosclerosis, and inflammation [1,2]. Seafood consumption has been encouraged as a source of n-3 PUFA, particularly eicosapentaenoic acid (EPA) and docosahexaenoic acid (DHA) [3]. Maternal intake of n-3 PUFA may lead to better neonatal outcomes such as reduced incidence of preterm birth [4,5,6]. Although many prenatal vitamins contain DHA, studies have suggested that maternal intake of fish, rather than supplements containing DHA, may decrease the likelihood of preterm birth [7]. However, the recommendations for fish consumption during pregnancy are complicated. Studies have linked mercury (Hg), a common metal found in many seafood products, to adverse birth outcomes such as preterm birth, reduced fetal growth, and birth defects [8,9]. Pregnant women have been advised to avoid or limit their consumption of certain types of seafood due to high Hg content to two servings of low Hg-containing seafood per week [6,10]. 

The potentially contradictory effects of seafood make reliable estimation of its consumption particularly important. Food frequency questionnaires (FFQs) are commonly used to assess dietary habits [11]. Many studies have been conducted to validate these results using biomarkers in blood, urine, and plasma compared to reported diets captured in FFQs [12,13,14,15,16]. Because seafood is a source of PUFAs, past studies have researched and found that the amount of n-3 PUFA in the blood is correlated to the reported intake of seafood that is high in n-3 PUFA [13,17,18]. Many seafood products are also high in Hg, and reported intake of these products has also been correlated with blood Hg levels [19,20]. 

Although these studies have concluded that FFQs are a valid way to record seafood consumption, little research has been conducted to validate reported dietary consumption among pregnant women. While past studies have found that there is a correlation between seafood consumption and blood Hg levels among pregnant women [21], as well as seafood consumption and n-3 PUFA [22,23], to our knowledge little research has been conducted that compares pregnant to nonpregnant women in the same population. In addition, seafood intake is of special interest to researchers studying Gulf Coast populations. Understanding seafood consumption of residents in this area is especially important among women of reproductive age, since the recent oil spill has led to concerns regarding reproduction and possibly adverse birth outcomes [24]. As part of the effort to understand the oil spill’s impact, the Gulf Resilience on Women’s Health (GROWH) study recruited pregnant and nonpregnant women of reproductive age in Southeastern Louisiana. In this study, we aim to compare seafood reported in the FFQ used in the GROWH study to blood levels of Hg and n-3 PUFAs in this population. We also aim to understand whether correlations differ between pregnant and nonpregnant women. 

## 2. Methods and Methods

### 2.1. GROWH Study

The Gulf Resilience on Women’s Health (GROWH) study was created in response to the Deepwater Horizon oil spill in order to study environmental exposures and its effects on reproductive age women. Recruitment began in 2011, and 1788 participants were recruited when recruitment ended in December 2016. Women were recruited from health facilities offering obstetrics and gynecological services, Women, Infants and Children (WIC) clinics, day care centers, and community events throughout Southeastern Louisiana. Eligible women were 18–45 years of age and living in Southeastern Louisiana during the Deepwater Horizon oil spill. Pregnant women were recruited only if they had a singleton gestation. During recruitment, women completed an in-person questionnaire and were asked to provide saliva and blood samples. Of the 1788 women who were recruited, 634 women provided a blood sample; the most common reason for not obtaining a blood sample was not wanting to undergo a needle stick. A metal-free (blue top) tube was used for blood collection. Women were also given a questionnaire to complete at the site or to take home with a postmarked envelope and to be returned via mail. The study was approved by the Institutional Review Board of Tulane University (IRB No. 239911).

### 2.2. Measurements

#### 2.2.1. Biomarkers

Non-fasting blood samples were taken from participants at the time of recruitment. Mercury (Hg) and n-3 PUFA were estimated from blood samples. Serum blood levels of n-3 fatty acids were obtained by determining serum alpha-linolenic acid (ALA, 18:3n-3c), erythrocyte eicosapentaenoic acid (EPA, 20:5n-3c), docosapentaenoic acid (DPA, 22:5n-3c), and docosahexaenoic acid (DHA, 22:6-3c). Levels of each type of n-3 fatty acids as well as the total n-3 PUFA were evaluated. 

Serum fatty acid content was analyzed at the Department of Nutrition, Harvard School of Public Health, Boston, MA, USA. Methods for fatty acid determination have been previously described by Baylin et al. [25]. Briefly, gas-liquid chromatography was used and peak retention times were identified by injecting known standards (NuCheck Prep, Elysium, MN, USA), using ChemStation A.08.03 software (Agilent Technologies, Santa Clara, CA, USA) for analysis. Sample processing and freezing does not affect the fatty acid measurements, as determined by the comparison of two pools of frozen and fresh samples and short- vs. long-term freezing. Calculated values for all the fatty acids studied were monitored continuously by analysis of a pooled control sample (indistinguishable from other study samples) run with each extraction and analysis batch. In general, peaks that were near the sensitivity limit (close to 0.10 of the total area) had larger calculated values.

Methylmercury concentration was analyzed at the Department of Veterinary Integrative Biosciences, Texas A&M University, College Station, TX, USA. Blood samples were analyzed for Hg by two methods, both of which employed atomic absorption for the detection the cold vapor atomic absorption spectroscopy (CVAAS) and the direct mercury analysis (DMA) or thermal decomposition method. Laboratory quality control samples ensured comparability across methods. Samples analyzed by CVAAS were first digested with trace metal grade nitric acid, hydrogen peroxide, and hydrochloric acid, and then diluted to volume with deionized water. Aliquots of digestate were analyzed on a Cetac Quicktrace 7600 instrument, in which Hg was liberated from the digest solution by chemical reduction to Hg^0^ using SnCl_2_ and purging with argon. Samples analyzed by DMA were pipetted into precleaned and precombusted sample boats and then analyzed on a Nippon MA-3000 instrument, in which samples were dried and combusted in a stream of O_2_. Combustion products were passed through a catalyst tube and Hg^0^ was trapped on a gold-coated column. After Hg was quantitatively trapped on the column, it was released by heating and Hg^0^ was measured in a pair of atomic absorption cells.

#### 2.2.2. Seafood Consumption

Fish consumption was estimated from the questionnaire using a previously-validated survey [26,27], adapted to the geographical area by focusing on the seafood available in the Southeastern Louisiana. Participants were asked their consumption on 55 fish and fish products, fish oil vitamins, and approximate serving size of fish per sitting. When asked about fish consumption, women were asked about their typical consumption of fish, canned seafood, or shellfish item using an ordered category scale (never, less than once per month, once per month, 2–3 times per month, 1–2 times per week, 3–4 times per week, 5–6 times per week, or once or more per day). High-Hg seafood was determined based on recommendations from the National Resources Defense Council, Louisiana Department of Health, and the North Carolina Department of Health and Human Services [28,29,30]. Seafood containing n-3 PUFA was identified based on the nutrition content listed in the United States Department of Agriculture (USDA) [31]. Approximate consumption of seafood per month in ounces was estimated by summing according to habitual intake and estimated serving size, for both seafood containing moderate to high amounts of Hg and seafood containing n-3 PUFAs.

### 2.3. Statistical Analysis

Because Hg blood levels and n-3 PUFAs were not distributed normally, logarithmic values were calculated for each of these measurements. Wilcoxon Signed Rank test was used to test the differences in the median amount of self-reported seafood between pregnant and nonpregnant women. In order to test the correlation between blood Hg and n-3 fatty acid levels, Spearman’s correlation coefficients were calculated comparing blood Hg and each n-3 PUFA, as well as total n-3 PUFAs, to total seafood consumption; these calculations were repeated stratified by pregnancy status. Linear regression models were also created with seafood consumption predicting blood Hg levels, each n-3 PUFA individually, and total n-3 PUFA. Crude and adjusted models for each model were created. We tested for interaction of pregnancy seafood consumption as well as possible interaction between time since oil spill (dichotomized by less than three years and more than three years) and reported seafood consumption. If no interaction was present, adjusted models included age and pregnancy status as covariates.

## 3. Results

Descriptive statistics of women included in the sample are presented in Table 1. Women in the study sample were predominately black (57%) and the majority of the sample reported an income that was less than $30,000 a year. Of the study sample, 27.2% of women were pregnant at the time of recruitment in the study. The median blood Hg levels were 1.66 μ/mL, and the median n-3 PUFA level was 3.66% of total fatty acids consumed. There was a significant difference in seafood consumed when comparing pregnant vs. nonpregnant women (Table 2), with pregnant women reporting consuming less seafood.

Hg blood levels and consumption of Hg-containing seafood were significantly correlated (r = 0.15, *p* = 0.01), and total n-3 fatty acid blood levels and seafood consumption was also significantly correlated (r = 0.12, *p* = 0.03) (Table 3). There was a significant correlation between EPA (r = 0.20), DPA (r = 0.11), and DHA (r = 0.16) and seafood consumption, but there was no significant correlation with ALA. When stratified by pregnancy status, only Hg was significantly correlated in both pregnant (r = 0.22) and nonpregnant (r = 0.10) women. Correlations for total n-3 fatty acids (r = 0.15) as well as with EPA (r = 0.21), DPA (r = 0.12) and DHA (r = 0.20), were higher in magnitude nonpregnant women (and statistically significant only in these women), although confidence intervals for the two groups overlapped. To further investigate the correlation between reported seafood consumption and blood levels of n-3 PUFA, we stratified correlations by trimester of pregnancy for all pregnant participants at the time of interview (Table 4). While the confidence intervals overlapped between all three trimesters, Hg was significantly correlated with reported consumption of Hg-containing fish for women in their first trimester (r = 0.66) and women in their second trimester (r = 0.26). Only for women in their first trimester was reported consumption of high-PUFA containing fish significantly correlated with any of the n-3 PUFA indicators (ALA r = 0.81). 

In linear regression models (Table 5 and Table 6), consumption of Hg-containing seafood was associated with Hg blood levels in both unadjusted and adjusted models. The highest quartile of seafood consumption was associated with an increase of blood Hg levels (adjusted β = 0.28). Total blood levels n-3 PUFA or ALA was not found to be significantly associated with seafood consumption of n-3 PUFA-containing seafood. Seafood consumption was predictive of EPA biomarkers in the highest quartile of consumption (adjusted β = 0.21). In the DPA and DHA models, there was an interaction with time. Women who reported their seafood intake in quartiles 2 (6–20 oz/month) and 3 (21–46.5 oz/month) and who had experienced the oil spill within the past three years tended to be less correlated with blood n-3 PUFA compared to those reporting in the first quartile (0−5.5 oz/month) and who had experienced the oil spill for more than three years later. Pregnancy status (not included in table) was associated in adjusted models with blood serum ALA (β = 0.28), EPA (β = −0.23), and DHA (β = 0.23), but we observed no interaction with seafood consumption and pregnancy status. 

## 4. Discussion

The results of the study suggest that, overall, self-reported seafood consumption is correlated with biomarkers for n-3 PUFA and Hg, however, the correlations are weak. Dietary questionnaires are known to have issues with error in self-report [32]. Similar studies evaluating the validity of FFQs and seafood consumption have found weak correlations when comparing blood serum samples of n-3 PUFA to seafood consumption [22,26,27,33]. A systematic review by Serra-Majem et al. on literature regarding biomarkers and n-3 PUFA found that, although some studies found strong correlations, EPA and DHA produced weaker correlations (r= 0.23–0.38 and r= 0.19–0.56, respectively) [18]. The correlations found in this study are slightly lower than the results reported by Serra-Majem et al. Our correlations are also slightly lower than comparable studies of pregnant women in regard to self-reported fish consumption and Hg levels [34]. 

It is also possible that concentrations of Hg and n-3 PUFA in seafood could have been affected by the Deepwater Horizon oil spill, which may make it difficult to compare this sample to other studies. Hg levels in fish in the Gulf waters tend to be higher compared to other geographic areas [35], and scientists were concerned that there could be an increase in levels of Hg found in seafood after the oil spill [36]. There is evidence, however, that the Hg levels found in seafood remained unchanged after the spill when compared to levels recorded before the spill [37]. Fatty acids in fish could have also been affected by the oil spill. Previous research has demonstrated that exposure to polycyclic aromatic hydrocarbons (that fish are exposed to in areas where oil extraction takes place) altered fatty acid composition [38].

We also found that time since the oil spill interacted with quartiles of reported seafood consumption in the DPA and DHA models. We found that the middle quartiles (those consuming between 20–46.5 oz/month of seafood) were less correlated with blood n-3 PUFA levels within three years of the oil spill compared to those who reported more than three years after the oil spill. This could be explained by inaccuracy of reporting seafood consumption in the middle quartiles of seafood consumption reporting. The presence of a disaster could have enhanced these inaccuracies in people who do not report eating high or low levels of seafood. Those who eat particularly high or low levels of seafood may be most accurate in their report, while the presence of a disaster could have enhanced inaccuracies in the people in the middle groups.

Pregnant women in our sample reported much less seafood consumption than nonpregnant women in this study. Lower seafood consumption among pregnant women has been found in other populations [39]. This lower consumption has been attributed by some researchers to guidelines suggesting limiting Hg-containing seafood during pregnancy [10]. A qualitative study identified that while some women indicate that they do not understand the health benefits of fish intake, issues of nausea and the cost of seafood are also barriers to fish consumption during pregnancy [40]. 

Pregnant women in this study had a higher correlation between reported Hg-containing seafood consumption compared to nonpregnant women, though the confidence intervals for these correlations overlapped and there did not appear to be an effect modification according to pregnancy status. Pregnant women having a higher correlation between reported Hg-containing seafood consumption and blood Hg levels could suggest that women better recall their intake of seafood that has moderate or high Hg content [21,34]. The Food and Drug Administration (FDA) recommends that pregnant women limit the amount of seafood to two 4-ounce servings per week [10], and this warning could mean that pregnant women are better at recalling their seafood intake if it contains higher levels of Hg. There is evidence that pregnant women have knowledge of this advice. Oken et al. found that pregnant women ate less fish after a 2001 national mercury advisory [41]. In studies conducted on pregnant women, FFQs were validated with biomarkers of Hg. 

Conversely, unlike nonpregnant women, pregnant women’s self-report of fish intake was not correlated with blood n-3 PUFA levels. The population recruited in this study were from areas affected by the Deepwater Horizon oil spill. It is possible that pregnant women may also be less inclined to report seafood that may have originated from oil spill-affected areas. In a previous analysis of this population, there was a significant decrease in seafood consumption reported after the oil spill compared to before the oil spill, and women were aware of possible dangers in consuming fish in Gulf waters [42]. Unlike mercury-containing seafood, pregnant women may not be able to recall n-3 PUFA-containing seafood because of the lack of awareness of these fish during pregnancy. Because pregnant women may be more concerned about their mercury consumption through seafood, they may be less cognizant of the fish they consume that does not contain mercury. Their lack of awareness could lead to pregnant women to falsely report their seafood consumption.

### Limitations

Blood samples and answers to FFQs were recorded cross-sectionally. This could produce biased answers, and blood levels may not be the best approximation for typical individual blood levels at that point in time. The blood samples were non-fasting, which could also have affected n-3 PUFA concentrations. We did not ask if seafood was frozen or fresh; if women ate primarily frozen seafood, then any assumptions that the seafood came from the gulf would not be correct. The oil spill also resulted in an increase in seafood prices which could have resulted in women eating cheaper seafood with higher mercury content, which could have also affected the results.

The study was conducted in Southeastern Louisiana and the population may not be reflective of other geographic areas. This is especially true in terms of seafood intake. The Environmental Protection Agency has reported that women of reproductive age each ate an average of 308.5 g of fish per month in 2010 [43], but GROWH Study participants report a much higher seafood consumption. The mean consumption of seafood in our study population was 1892 g per month, while pregnant women still reported 1219 g per month. This indicates that women could potentially receive much of their PUFA from seafood but are also potentially exposed to high levels of Hg. However, we do expect the amount of seafood intake to be representative of many of coastal communities in the southern and southeastern United States.

## 5. Conclusions

This is the first study, to our knowledge, that compares the validity of biomarkers for seafood intake for pregnant and nonpregnant women. The population studied tended to consume more seafood and were from an area recovering from a large-scale oil spill that may have polluted much of the seafood consumed. Our study confirmed that, as has been found in other populations, pregnant women in this study population eat less seafood compared to nonpregnant women. There were also significant correlations between blood Hg levels and reported moderate to high Hg-containing seafood, and the correlation was stronger in pregnant vs. nonpregnant women. However, only nonpregnant women reported significant correlations for n-3 PUFAs compared to pregnant women. While pregnant women may be able to better report Hg-containing seafood, they may be less inclined to report overall fish consumption, resulting in a less valid self-report of n-3 PUFA intake. 

## Figures and Tables

**Table 1:** Descriptive characteristics of Gulf Resilience on Women’s Health (GROWH) participants, *n* = 669.

Characteristic	*n*	%
Race		
White	189	28.3
Black	381	57.0
Other	46	6.9
Missing	53	7.9
Age (years)		
18–19	179	26.8
20–24	177	26.5
25–35	116	17.3
35+	124	18.5
Missing	73	10.9
Income (USD)		
<15 k	256	38.3
15–30 k	212	31.7
>30 k	124	18.5
Missing	77	11.5
Pregnancy status		
Pregnant	182	27.2
Not pregnant	487	72.8
Missing	0	0
Region		
Coast	134	20.0
Inland	485	72.5
Missing	50	7.5
Fish Oil		
Yes	53	7.9
No	390	58.3
Don’t know/missing	226	33.7
**Blood Levels**	**Median**	**25th and 75th Percentile**
Hg (ng/mL)	1.66	1.23, 3.49
Total n-3 PUFA (% total FA)	3.66	1.68, 5.10
ALA (% total FA)	0.15	0.11, 0.20
EPA (% total FA)	0.22	0.13, 0.41
DPA (% total FA)	1.05	0.50, 1.50
DHA	1.93	0.86, 2.91
**Self-Reported Seafood Consumption (Ounces per Month)**	**Median**	**25th and 75th Percentile**
Hg	9.0	2.5, 25
Total n-3 PUFA	20	6, 47.5

**Table 2:** Comparison of reported seafood consumption of seafood containing mercury, n-3 PUFA between pregnant and nonpregnant women in ounces consumed per month *.

Seafood Consumption	Pregnant			Non-Pregnant		
	Median	25th Percentile	75th Percentile	Median	25th Percentile	75th Percentile
Moderate Hg seafood	5	1	18	10.5	3	27.5
High n-3 PUFA seafood	12.5	3.5	32.5	22.5	7.5	56.0

* significant at *p* = 0.05 level using Wilcoxon Signed Rank.

**Table 3:** Spearman’s correlation coefficients comparing reported seafood consumption and blood levels for Hg (*n* = 549) and n-3 PUFAs (*n* = 356).

Blood Level	Overall		Pregnant		Not Pregnant	
	r	95% CI	r	95% CI	r	95% CI
Hg	0.15 *	0.07, 0.23	0.22 *	0.06, 0.37	0.10 *	0.01, 0.20
Total n-3 PUFA	0.12 *	0.02, 0.22	−0.01	−0.24, 0.23	0.15 *	0.03, 0.26
ALA	0.00	−0.10, 0.11	−0.10	−0.33, 0.13	0.05	−0.06, 0.17
EPA	0.20 *	0.10, 0.30	0.06	−0.17, 0.29	0.21 *	0.10, 0.32
DPA	0.11 *	0.01, 0.23	0.03	−0.20, 0.27	0.12 *	0.00, 0.23
DHA	0.16 *	0.06, 0.26	0.05	−0.18, 0.28	0.20 *	0.09, 0.31

* Significant at *p* = 0.05.

**Table 4:** Spearman’s correlation coefficients comparing reported seafood consumption and blood levels stratified by trimester.

Blood Level	Trimester 1 *n* = 22		Trimester 2 *n* = 66		Trimester 3 *n* = 71	
	r	95% CI	r	95% CI	r	95% CI
Hg	0.66 *	0.27, 0.86	0.26 *	0.01, 0.48	0.13	−0.12, 0.36
Total n-3 PUFA	0.06	−0.79, 0.83	0.08	−0.35, 0.49	−0.01	−0.33, 0.31
ALA	0.81 *	0.00, 0.98	0.05	−0.37, 0.42	−0.22	−0.51, 0.11
EPA	0.32	−0.66, 0.90	−0.01	−0.43, 0.42	0.12	−0.21, 0.43
DPA	−0.41	−0.92, 0.69	0.03	−0.39, 0.45	0.12	−0.21, 0.43
DHA	0.06	−0.79, 0.83	0.10	−0.34, 0.50	0.12	−0.21, 0.43

* Significant at *p*= 0.05.

**Table 5:** Linear regression models modeling for seafood consumption (measured in quartiles of ounces of seafood consumption per month) as a predictor for blood levels of Hg and total n-3 PUFA.

Model	Unadjusted			Adjusted		
	β	95% CI	*p*-Value	β	95% CI	*p*-Value
Hg *						
Seafood Consumption **			0.01			0.03
Q1	Ref			Ref		
Q2	0.11	−0.09, 0.30		0.05	−0.16, 0.25	
Q3	0.12	−0.08, 0.32		0.09	−0.11, 0.29	
Q4	0.34	0.15, 0.53		0.28	0.08, 0.48	
Total n-3 PUFA *						
Seafood Consumption ***			0.07			0.12
Q1	Ref			Ref		
Q2	−0.12	−0.31, 0.07		−0.16	−0.35, 0.03	
Q3	−0.03	−0.22, 0.17		−0.06	−0.26, 0.14	
Q4	0.12	−0.07, 0.31		0.07	−0.13, 0.26	

* Adjusted for pregnancy status and age; ** Q1 = 0–2.5 oz/month, Q2 = 3–9 oz/month, Q3 = 10–24.5 oz/month, Q4 = 25.5–595 oz/month; *** Q1 = 0–5.5 oz/month, Q2 = 6–20 oz/month, Q3 = 21–46.5 oz/month, Q4 = 47.5–714 oz/month.

**Table 6:** Linear regression models modeling for seafood consumption (measured in quartiles of ounces of seafood consumption per month *) as a predictor for blood levels of ALA, EPA, DPA, and DHA.

Model	Unadjusted			Adjusted		
	β	95% CI	*p*-Value	β	95% CI	*p*-Value
ALA **						
Seafood Consumption			0.36			0.32
Q1	Ref			Ref		
Q2	−0.13	−0.36, 0.10		−0.12	−0.36, 0.13	
Q3	−0.15	−0.40, 0.08		−0.17	−0.43, 0.08	
Q4	−0.21	−0.44, 0.03		−0.23	−0.47, 0.02	
EPA **						
Seafood Consumption			0.004			0.05
Q1	Ref			Ref		
Q2	0.07	−0.11, 0.24		0.01	−0.17, 0.18	
Q3	0.13	−0.06, 0.31		0.08	−0.09, 0.26	
Q4	0.31	0.13, 0.49		0.21	0.04, 0.38	
DPA ***						
Seafood Consumption			0.20			0.14
Q1	Ref			Ref		
Q2	−0.03	−0.24, 0.18		0.12	−0.12, 0.37	
Q3	0.07	−0.15, 0.30		0.29	−0.03, 0.54	
Q4	0.18	−0.03, 0.40		0.22	−0.03, 0.46	
Consumption/Time interaction						0.002
Q1 × Time	-	-		Ref		
Q2 × Time	-	-		−0.72	−1.23, −0.21	
Q3 × Time	-	-		−0.95	−1.47, −0.43	
Q4 × Time	-	-		−0.33	−0.85, 0.19	
DHA ***						
Seafood Consumption			0.01			0.17
Q1	Ref			Ref		
Q2	−0.08	−0.29, 0.13		−0.13	−0.23, 0.26	
Q3	0.05	−0.17, 0.28		0.16	−0.09, 0.41	
Q4	0.27	0.06, 0.49		0.24	−0.01, 0.48	
Consumption/Time interaction						0.02
Q1 × Time	-	-		Ref		
Q2 × Time	-	-		−0.61	−1.12, −0.10	
Q3 × Time	-	-		−0.60	−1.12, −0.08	
Q4 × Time	-	-		−0.07	−0.59, 0.45	

* Q1 = 0–5.5 oz/month, Q2 = 6–20 oz/month, Q3 = 21–46.5 oz/month, Q4 = 47.5–714 oz/month; ** Adjusted for pregnancy status and age; *** Adjusted for pregnancy status and age with time since oil spill/seafood consumption interaction.
